# Supplementary material for: Adoptive Transfer of CX3CR1-Transduced Tregs Homing to the Forebrain in Lipopolysaccharide-Induced Neuroinflammation and 3xTg Alzheimer’s Disease Models
Source: Int J Mol Sci. 2024 Dec 21;25(24):13682. doi: 10.3390/ijms252413682 (PMC11727661; doi:10.3390/ijms252413682)
Supplement: Supplementary file 1 [file ijms-25-13682-s001.zip › ijms-3372022-supplementary.pdf]

A

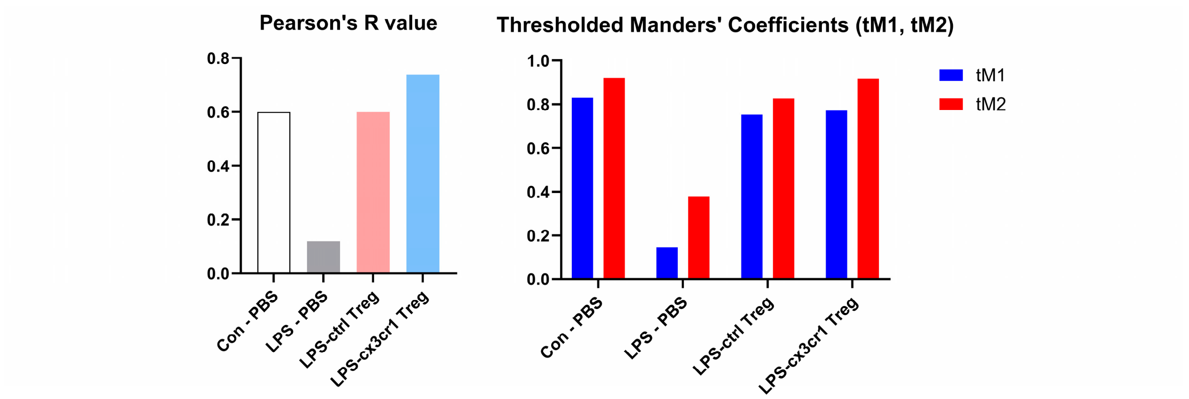

B

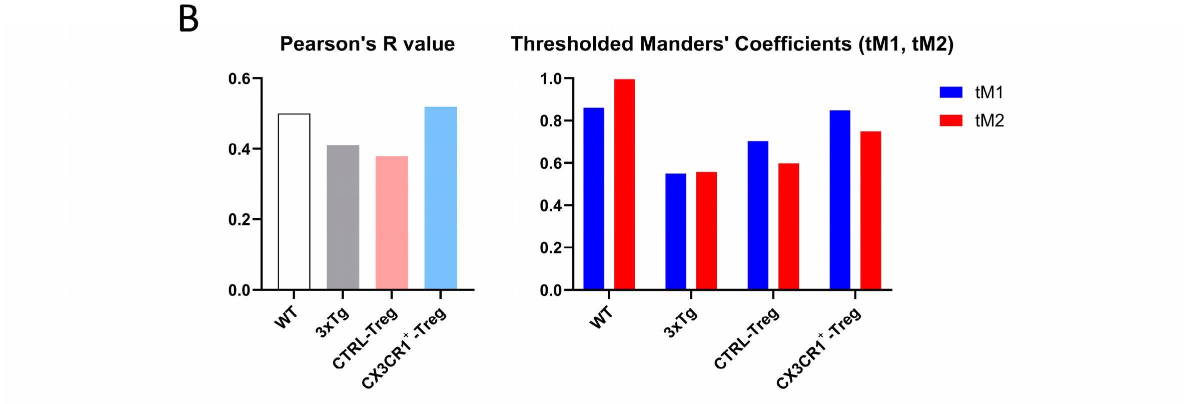

Supplementary Figure S1: To assess the extent of Iba1 translocation into the nucleus, co-localization analysis of Iba1 (red) and nuclear signals (blue, DAPI) was conducted in Figure 3C and Figure 7C using ImageJ/FIJI software. Channel 1 was designated as blue. Pearson's R values (non-threshold) and Thresholded Mlanders' Coefficients (tM1 and tM2) were applied to quantify the degree of co-localization.
